# Prediction of the Cohesion Energy, Shear Modulus and Hardness of Single-Phase Metals and High-Entropy Alloys

**DOI:** 10.3390/ma17112728

**Published:** 2024-06-04

**Authors:** Ottó K. Temesi, Lajos K. Varga, Nguyen Q. Chinh, Levente Vitos

**Affiliations:** 1 H-ION Kft., Konkoly-Thege Miklós út 29-33, 1121 Budapest, Hungary; otto.temesi@h-ion.hu; 2 SMARTUS Zrt., Gyár utca 2, 2040 Budaörs, Hungary; 3 Department of Materials Physics, Eötvös Loránd University, Pázmány Péter Sétány 1/A, 1117 Budapest, Hungary; chinh@metal.elta.hu; 4 HUN-REN Wigner Research Centre for Physics, P.O. Box 49, 1525 Budapest, Hungary; leveute@kth.se; 5 Department of Materials Science and Engineering, Royal Institute of Technology, SE-100 44 Stockholm, Sweden

**Keywords:** high-entropy alloys, compositions design, shear modulus, cohesion energy, hardness

## Abstract

In order to facilitate the prediction of some physical properties, we propose several simple formulas based on two parameters only, the metallic valence and metallic atomic radii. Knowing the composition, for single-phase alloys, the average parameters can be calculated by the rule of mixture. The input parameters can be obtained from tabulated databases. Adopting from the literature the results of Coulomb crystal model for metals and single-phase high-entropy alloys, we have derived formulas for the shear modulus (G) and the cohesion energy (E_coh_). Based on these parameters separately, we set up two formulas to estimate the hardness in the case of pure metals. For single-phase (solid-solution) HEAs, by simplifying the Maresca and Curtin model, we obtained a formula for estimating the hardness, which takes into account the atomic misfit in addition to G. The maximal hardness for single-phase HEA is approximately 600 kg/mm^2^ and is obtained for a composition with a valence electron concentration of approximately 6 ÷ 7.

## 1. Introduction

The process of designing materials from the first principles is not usually a straight-forward or simple one. It requires large computer capacities and selection between the competing models [1]. Machine learning [2], as a branch of artificial intelligence, can help overcome the shortcomings of long-term experiments and computational simulations and effectively shorten the design of new alloys. However, we lack a deeper understanding of cause and effect relationships. This is why simple phenomenological connections are more effective and reveal the physical content as well.

The physical properties of alloys are defined at the atomic level. This is why one is tempted to work with semi-empirical models based on atomic valences and bond distances. Such papers have been published on volumetric lattice energy [3,4], bond ionicity [5] and the melting point [6].

In order to facilitate the design of high-entropy alloys (HEAs), a simple approximate electrostatic model is presented using only the atomic valences and atomic radii. In this model, a metal can be considered as a “one-component plasma” (OCP) [7], which is a system of positive point charge embedded in a uniform distributed compensating negative charge sea of valence electrons. The OCP model is very popular nowadays because it can be applied in different systems like charge-stabilized colloids [8] and dense neutron stars [9,10].

When the ratio, Γ, of electrostatic energy to thermal energy, exceeds a value of Γ = 175, OCP freezes into a body-centered cubic (bcc) Coulomb crystal, which can be treated classically [7]. It was shown in the literature [10,11,12] that the matter in the cores of white dwarfs and the crusts of neutron stars can be modeled as a Coulomb crystal of fully pressure-ionized atomic nuclei with a nearly incompressible background of strongly degenerate electrons. We suppose that the elastic properties of their matter can be treated as similar to those of metals where the exchange interaction of the electrons is neglected as a first approximation in the calculation of the shear modulus. This is the basic supposition of the present work.

It is worth mentioning that in 1962, Keyes [13] presented a conjecture concerning the electrostatic origin of the elastic moduli. Based on dimensional analysis, he proposed a normalization factor, which is equal to the electrostatic energy per unit volume: e^2^/R^4^, where e is the electric charge and R is the atomic radius. Based on the electrostatic origin of the shear (and also of bulk) modulus, a formula for the shear modulus, G, can be simply derived as the second derivative of electrostatic energy. J. Gilman obtained, by a “back-on -the envelope” calculation [14], the following estimation formula.

(1) $$G=\frac{3}{32\pi }\frac{e^{2}}{R^{4}},$$

where *e* is the charge of the electron and *R* is the atomic radius.

Formula (1), however, is a rough approximation because the valence of the metal is not taken into account.

The aim of this work is to use the results published in the literature [10,11,12] on the Coulomb crystal to calculate the shear modulus, *G*, and then, based on own considerations, to extend the Coulomb crystal model calculations to the cohesion energy, *E_coh_*, of single-phase metal alloys (pure metals and high-entropy alloys). Then, we set up two formulas to estimate the hardness based on *G* and *E_coh_* and compare the goodness of the estimation. The formulas are tested first on pure metals and then it is applied for HEAs compositions. In the case of multicomponent single-phase HEAs, solid-solution hardening will be discussed in addition to the result obtained by the rule of mixture applied to the component elements.

## 2. New Approaches on Estimation of Physical Properties

### 2.1. Prediction of the Shear Modulus

Modelling the metal with a Coulomb crystal, we a going to calculate the shear moduli (and later the cohesive energy) applying a severe simplification based on the “equivalent (or effective) atom” [11]. This can be performed for an isotropic alloy where all of the atoms in the Coulomb crystal are the same. The calculations are based on electrostatics determining the potential energy of the equivalent atom. This equivalent atom is actually a positive nucleus immersed in a uniform electron cloud of mean square radius, R_m_.

For such a Coulomb lattice, the elastic constants were calculated by Baiko [10,11], and using the Voight, Reuss and Hill approximations [12], the effective shear modulus G is given [12] as:

(2) $$G_{eff}=0.3462\frac{n_{N}e^{2}Z^{2}}{2a}$$

where $n_{N}=\frac{3}{2\times 4\pi {R^{3}}_{ws}}$ is the number density with two atoms/cell, and *a* is the length of the side of the cubic cell containing two atoms, *n_N_* is the number density of atoms, *Z* is the valence number of the positive ions, *R_ws_* is the Wigner–Seitz atomic radius and *e* is the elementary charge. Formula (2) should be multiplied with 9 × 10^9^ In order to get the result in SI units, and the Wigner–Seitz radius replaced by atomic metallic radius for BCC system R_m_ = 0.8793 × R_ws_.

Inserting the numerical factors after some algebra, we obtain a formula which depends on two tabulated properties only, the valence *Z* and the metallic atomic radius, *R_m_*, which are easily accessible at [15]:

(3) $$G_{eff}=14\frac{Z^{2}}{R_{m}^{4}},$$

where *R_m_* is in Å and *Z* is the valence and the unit for *G_eff_* is GPa.

It is the basic observation of this paper that Formula (2) calculated for neutron stars is equally valid for the polycrystalline metals—just the number density must be matched to the condensed matter data and the value of *Z* is not the proton number of ionic nuclei (which has lost all its electrons) but the number of valence electrons given in the common electron sea background. The exact number of valence electrons depends on the property under calculations. For chemical reactions and corrosion behavior, the chemical valence should be applied (in general the sum of the electrons on the last s-p shell); for the cohesion energy, however, where the bonding character should be accentuated, in addition to the outer shell *sp* electrons, the uncompensated *d* electrons must be taken into consideration. We considered that the valence of Miedema, *Z_BM_*, calculated from the bulk modulus, *B*, is the most appropriate to calculate the shear modulus. Miedema valences were calculated from the tabulated data of *B* and *V_m_* using the following equation [16]:

(4) $$n=10^{-2}\times 6.748\times \sqrt{\frac{B}{V_{m}}}$$

(5) $$Z_{BM}=n\cdot V_{ws}=n\times 1.666\times V_{m}$$

where the molar volume, *V_m_* (cm^3^), is calculated from the atomic mass (*A*) and density *ρ* (*V_m_ = A*/*ρ*) and the atomic volume $V_{ws}(A{{}^{\circ}}^{3})=1.66\cdot V_{m}(cm^{3})$.

It should be mentioned that the valence *Z* is used as the valence electron number per atom (*e*/*a*), the contribution of each atom to the shared electron sea and should be distinguished from the valence electron count (VEC), which comprises the electrons on the outer electron shells (s-p-d), a part of them, participates only conditionally in the metallic bond.

The *R_m_* and *Z* data of the elements used in practice according to the three most common crystal lattice symmetries are collected in Table 1, together with the calculated shear modulus values using Formula (3).

**Figure 1:**
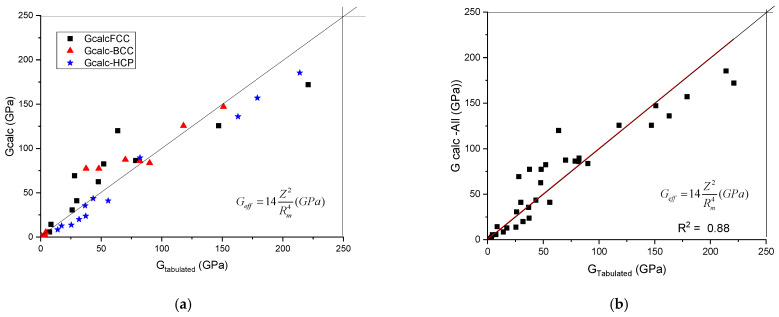
A plot of the calculated shear modulus against the tabulated (accepted) values. (**a**) distinguished according to crystal structure, (**b**) plotted together to establish the goodness of fit.

The calculated shear modulus, G_calc_, as a function of tabulated ones is presented in Figure 1. It can be seen that the data line up along the first angle bisector. No separation of the data as a function of crystalline structure was found. To establish the quality of the goodness of fit, we treated the data belonging to the three crystal symmetries together (see Figure 1b) and a R^2^ value of 0.88 was obtained.

### 2.2. Estimation of the Cohesion Energy

We have adopted a simple metal model, which is composed of positive ionic cores distributed in a regular crystallographic lattice and the negative sea of valence electrons. The Coulombian interaction takes place between the cation with a charge +Ze and the partial number of surrounding electrons, –e/n. Taking a coordination number of 12, attractive interaction will be manifested between 6 ion pairs only at a given moment:

(6) $$E_{coh}=9\times 10^{9}\frac{M_{d}}{6}\frac{Ze^{2}}{d}=4.12\frac{Z}{d};$$

where d is the distance between the atoms in Angstrom, d = 2R_m_, R_m_ is the metallic atomic radius in Å, and then the unit of the cohesion energy is eV.

*M_d_* is the Madelung constant summing up the effect of surrounding atoms. Its value is approximately 1.716 [17], and it was used to calculate the numerical factor in Formula (6).

For comparison, let us recall the formulas valid for ionic binding [17]:

(7) $$E_{c}=9\times 10^{9}M_{d}\frac{Z_{1}\cdot Z_{2}\cdot e^{2}}{d}=24.71\frac{Z_{1}Z_{2}}{d},$$

where *M_d_* = 1.716 is the Madelung number and d is the distance between the atoms in Angstrom.

We find differences in two points. First, for metals, the binding energy is proportional to the valence, whereas for ionic binding to the product of valences; and second, for metals, the Madelung number is divided by 6, because from 12 near neighbors, only 6 pairs can be formed and, at a given moment, only 1/6 parts of interactions is effective. Although the higher valence somewhat compensates for this 1/6 multiplier, in general, the metallic bond is weaker than the ionic one.

It is to be observed that the cohesion energy formula does not contain free parameters for fitting the results. Certainly, these formulas are only approximate. The calculated data of Equation (6) are systematically 15–20% greater than the accepted ones. The reason for this is presumably that the influence of the environment was taken into account in an unsatisfactory way, that is, the Madelung constant must be reduced by a factor of 0.82. Finally, the formula recommended for alloy design is:

(8) $$E_{coh}=1.7\frac{Z}{R_{m}};$$

**Figure 2:**
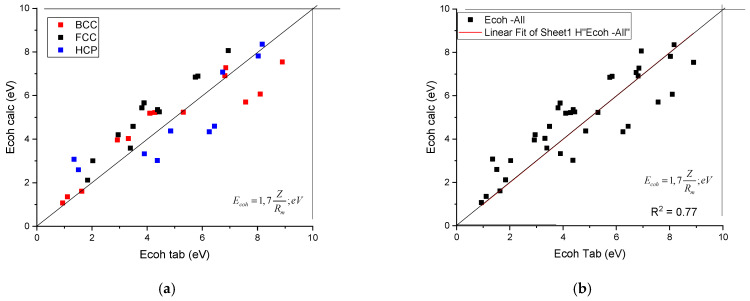
A plot of the calculated cohesion energy against the tabulated ones. (**a**) distinguished according to crystal structure, (**b**) plotted together to establish the goodness of fit.

Using the Z and R_m_ data collected in Table 1, the calculated cohesion energies as a function of tabulated ones are shown in Figure 2. It can be seen that the data points line up along the first angle bisector, with an R^2^ = 0.77. No separation of the data as a function of crystalline structure can be observed.

It is worth mentioning that there is a strong correlation between the cohesion energy and the melting temperature:

$$E_{coh}=0.24\cdot T_{m}$$

**Figure 3:**
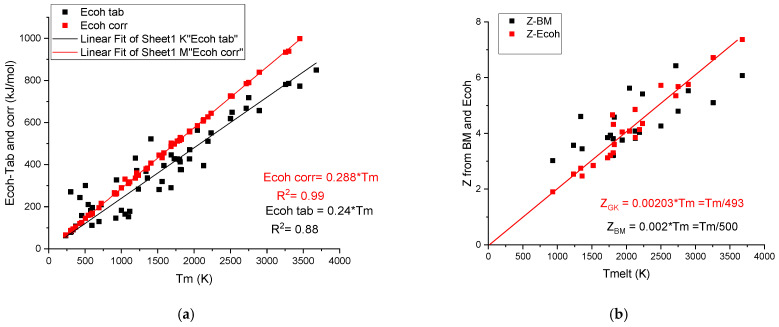
(**a**) The linear corrected (GK) and scattered uncorrected (Tab) cohesion energy data versus melting temperature. The difference increases with the increasing melting temperature. (**b**) The calculated valences Z-Bm and Z-Ecoh values versus melting temperature.

Figure 3a shows a strong correlation with R^2^ = 0.88 between the tabulated values and *T_m_*. G. Kaptay [18] applied a correction to the tabulated cohesion energy data, obtaining a perfect linear correlation:

$$E_{coh-corr}=0.288\cdot T_{m}$$

The corrected values of cohesion energies, *E_coh-corr_*, are as an average 0.288/0.24 = 1.18-fold larger than the average uncorrected (tabulated) values. This correction beneficially increases the accuracy of the prediction when we estimate hardness using cohesive energy.

It is worth noting that based on Equation (8) of the cohesion energy, an effective valency can be determined, which is also a linear function of the melting temperature. Valences, Z_GK_, determined using the corrected cohesion energies [18], are less scattering (see Figure 3b) around the fitting line (Z_GK_ = 0.00203*Tm) than the valences determined based on bulk modulus, Z_BM_, using the Miedema model (Equation (4)). The average Z is approximately Tm/500 in both cases (see Table 2). The large metallic valence number, Z, for transition metal elements shows that in addition to the s-p electrons, the d electrons participate also in the common valence electron sea. As to our knowledge, the above presented correlation between the metallic valence and the melting temperature was not yet discussed in the literature.

### 2.3. Estimation of HV from the Cohesion Energy, Ecoh

In order to predict and tailor the hardness of single-phase HEAs we need a prior relationship, with calculable or measurable parameters. We first examine how to derive hardness from cohesive energy and then from elastic moduli.

Based on dimensional analyses, we suggest that the HV is proportional to the cohesion energy multiplied with the electron density, n = Z/V_m_, where Z is the metallic valence and V_m_ is the molar volume. In this way, in addition to the unpaired d electrons, the sp electrons are taken into account as well in determining of hardness. The phenomenological equation is:

(9) $$HV_{calc}=C\cdot E_{c}\frac{Z}{V_{m}},$$

where C is a constant to be determined, Z is the valence, Ec is in kJ/mol (1 eV/atom = 96.485 kJ/mol) and V_m_ is in cm^3^, then the unit of the calculated hardness, HV calc., is in GPa.

**Figure 4:**
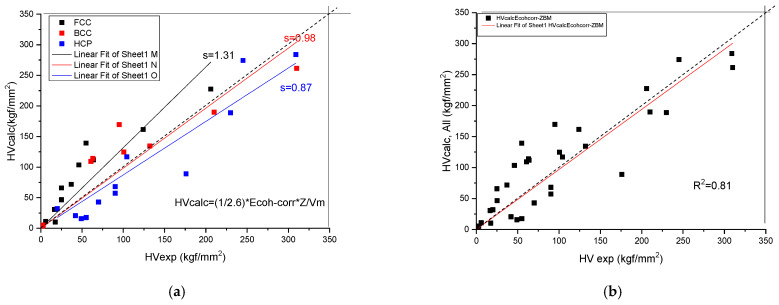
Correlation of the measured and calculated Vickers Hardness based on Formula (9) for pure metals. (**a**) distinguished according to crystal structure, (**b**) plotted together to establish the goodness of fit.

We have found (see Figure 4 and Table 3) a rather good correlation between the calculated and measured values although the measured hardness values published in the literature [19] show a great range. Unfortunately, the tabulated Ec and Z values show a great range also. This is why it is necessary for a review article to collect and comment on the valence and atomic radii data published in the literature so far.

A normalizing factor C = 1/(100*2.6) adjusts the values to GPa and finally the dimensions to the measured values is obtained by multiplying by 100, because 1 GPa = 100 kgf/mm^2^. For high-entropy alloys, all three parameters (Ec, Z and V_m_) can be calculated as a weighted average of the elemental values. Equation (9) permits estimation of the low bound for hardness of any single-phase alloy.

Formula (9) is a good addition to the literature [19], where various correlations between hardness and different elastic moduli or a combination of them have been published. However, the best correlation can be achieved with the shear modulus, G, which can be understood by the creation and movement of the dislocation, which is not facilitated by the volume change, but by the shear deformation.

### 2.4. Estimation of Hardness, HV, from the Shear Modulus, G

It was shown [19] that for brittle alloys (BMG and compounds), where the plastic deformation is negligible compared to the elastic one, there is a direct relationship between HV and G:

(10) HV=0.151·G.

For pure metals and ductile HEAs, however, an order of magnitude greater calculated hardness results from Equation (10). This is why a new relationship is necessary to calculate the hardness based on elastic moduli. We start from the formula given in the literature [20]:

(11) $$HV=\frac{G}{B}\frac{E}{6}\frac{1-2\nu }{1+\nu },$$

where we change E to G and apply the well-known relations valid for isotropic materials E=3B(1−2ν) and E=2G(1+ν) and we obtain the hardness as a function of G and of Pugh [21] (ductile–brittle) parameter, G/B. Not that a sample is ductile for G/B < 0.6, and brittle for G/B > 0.6.

(12) $$HV=\frac{G}{B}\frac{G}{6}\frac{1-2\nu }{1+\nu }=\frac{1}{9}{\left(\frac{G}{B}\right)}^{2}\cdot G,$$

**Figure 5:**
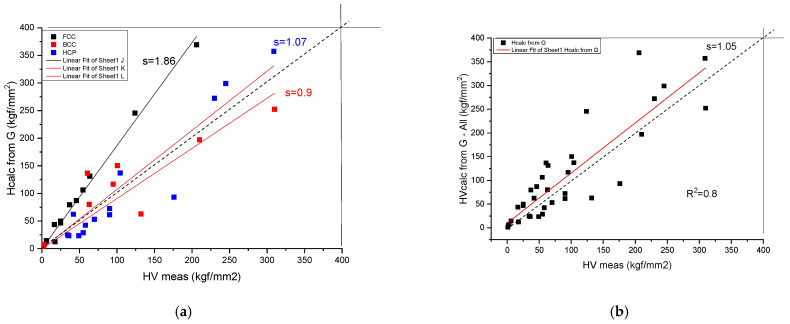
Correlation of the measured and calculated Vickers Hardness based on Formula (14) for pure metals. (**a**) distinguished according to crystal structure, (**b**) plotted together to establish the goodness of fit.

For a representative value of G/B = 3/8, the result is:

(13) HV=0.0156·G

which matches the order of magnitude of the experimental HV values. For more exact matching, a fitting coefficient, C, is determined (see Figure 5):

(14) HV=C·0.0156·G

The slope in Figure 5b is *s* = 1.0760, so the improved relationship is:

(15) $${HV}_{calc.}=0.0167\cdot G,$$

where the units at both sides are in GPA. In order to obtain HV_calc_ in kgf/mm^2^, one has to apply a factor of 100. The calculated values for pure metals are collected in Table 3. 

### 2.5. Estimation of Hardness of Single-Phase HEAs

The basic parameters, the atomic radii and valences, will be calculated using the rule of mixture (ROM), where the weighted average is calculated against the concentration of the elements, as

(16) $$R=\sum_{i}C_{i}R_{i}\;\mathrm{and}\;Z=\sum_{i}C_{i}Z_{i}$$

In order to estimate the hardness of single-phase HEAs, we need a new parameter, the atomic size misfit, which is the mean squared deviation of the atomic size, *R_a_* or *R_ws_*:

(17) $$\delta_{a}=100\sqrt{c_{i}{\left(\frac{R_{ai}-\bar{R_{a}}}{\bar{R_{a}}}\right)}^{2}}$$

We have to distinguish between volume per atom, *V_ws_* = *V_m_*/*N_A_*, and atomic volume, *V_a_*, where from the corresponding Wigner–Seitz, *R_ws_* and atomic, *R_a_*, radii can be determined. It should be emphasized that whereas *R_ws_* is independent of crystalline structure, the *R_a_* depends on the structure because the atomic radii is defined as half of the distance between the atoms. The relations between the *R_a_* and the lattice constant, *a*, for the relevant three crystalline structures of the single-phase HEAs are the following: Ra = a*√3/4 for BCC, Ra = a*√2/4 for FCC and Ra = a/2 for HCP. The volume per unit cell is *a*^3^ for BCC and FCC and c*a^3^*3√3/2 for HCP structures, while the number of atoms per unit cell is 2 for BCC, 4 for FCC and 6 for HCP structures, respectively. Performing simple calculations, we find *R_a_* = 0.8793*R_ws_ for BCC, *R_a_* = 0.9046 * *R_ws_* for FCC and *R_a_* = 0.9117 * *R_ws_* for HCP structures.

The expression of atomic size as *R_a_* and *R_ws_* is important when one calculates the atomic misfit *δa* and *δws*. These two misfit parameters are the same when the alloy and all the constituents belong to the same crystalline structure. In a number of cases, however, there are constituents with different structures like the HCP Ti-Zr-Hf elements in BCC refractory alloys or the FCC Al in BCC transition metal alloys. In such a case, we have to recalculate the *R_a_* parameter of the element from the structure-insensitive Rws using the above formulas and then we will obtain the same misfit parameters, δ*a* = *δws*. For example, for HCP titanium, we have from the tabulated data: *R_ws_* = 1.6145 Å and *R_a_* = 1.4707 Å. In BCC alloy, we have to consider *R_a_* = 1.635 Å (and a = 3.27 Å) for calculation of the atomic misfit.

The shear modulus <*G*> and the cohesion energy <*E_coh_*> will be calculated with ROM and with the Equations (3) and (8), respectively, using the weighted averaged *R* and *Z* values from Equation (16). The match of the data is acceptable. However, the calculated hardness values from *G* (Equation (15)) and from *E_coh_* (Equation (9)) are approximately 3–4-smaller than the measured ones (see Table 4). It is considered that this discrepancy can be attributed mainly to solid-solution hardening (SSH) as the other possible contributions like the grain boundary, second phase and precipitation strengthening will be neglected for the as-cast alloy samples.

SSH originates from lattice distortion, which is characterized by the atomic misfit due to the different elements in the multicomponent HEAs. A number of papers are dealing with numerical calculations of the yield stress (YS) and of the Vickers Hardness (HV = 3*YS) of solid-solution hardened alloys [22,23,24]. However, the scaling factor and the ratio of edge and screw dislocations are rather arbitrarily estimated, although the atomic misfit and elastic modulus misfit are accurately calculated. Therefore, we dare to make a reasonable simplification in the equations published in the literature for predicting the hardness due to solid-solution hardening.

Starting from Varennes’s concepts [22], Maresca and Curtin [24] developed a model applying the material constants available from the literature. They obtained the yield stress contribution of solid-solution hardening (SSH) as:

(18) $$\sigma_{y}=0.0407\alpha^{-1/3}G{\left(\frac{1+\nu }{1-\nu }\right)}^{4/3}{\left(\sum_{i}\frac{c_{i}\Delta V_{i}^{2}}{b^{6}}\right)}^{2/3},$$

where “G” and “ν” are the isotropic alloy elastic constants (shear modulus and Poison constant) and “*b*” is the alloy Burgers vector, calculated using Vegard’s law to determine the alloy volume (b = (4 V)^1/3^/(1/2)^0.5^). The computed misfit volumes closely follow Vegard’s law, Δ*V_i_* = *V_i_* − <*V>*, where <*V>* = Σ*c_i_V_i_* is the alloy atomic volume and *V_i_* the elemental BCC atomic volumes. “α” is the line tension parameter, α = 1/12.

The term containing the Poisson factor varies between 1.5 and 3. Let us take the most representative value of 2.5, and then the numerical factors can be simplified to

(19) $$\sigma_{y}=0.234\cdot G\cdot {\left(\sum_{i}\frac{c_{i}\Delta V_{i}^{2}}{b^{6}}\right)}^{2/3},$$

The sum in parenthesis can be written as $\sum_{i}c_{i}\cdot \Delta V_{i}^{2}=9\cdot {\bar{V}}^{2}\cdot \delta_{w}^{2}$, where *δ_w_* is the atomic size misfit expressed in terms of atomic radii.

Finally, we arrived to a formula which contains two parameters of the HEA, the averaged shear modulus, G, and the atomic misfit:

(20) $$\sigma_{y}=0.637\cdot G\cdot \delta_{w}^{4/3}.$$

The hardness increase due to solid-solution hardening can be estimated as

*HV* = 3*σ_y_*. (21)

The predicted hardness will be quantified as the sum of two contributions: the starting hardness of constituents calculated with the rule of mixture:

HV_base_ = Σc_i_HV_i_ (22)

and the contribution from solid-solution hardening given by Equation (22):

(23) $${\mathrm{HV}}_{\mathrm{calc}}={\mathrm{HV}}_{\mathrm{base}}+K\cdot G\cdot \delta_{w}^{4/3}$$

Creating a database from the literature, it turns out that for the majority of cases, *HV_base_* = 60 ± 20 and the fitted value for proportionality constant, K = 0.7, as long as the unit of G is in GPa.

As a result of all these approximations, the calculated value for the hardness of single- phase HEAs is:

(24) $${\mathrm{HV}}_{\mathrm{calc}}=60+0.7\cdot G\cdot \delta_{w}^{4/3}$$

**Figure 6:**
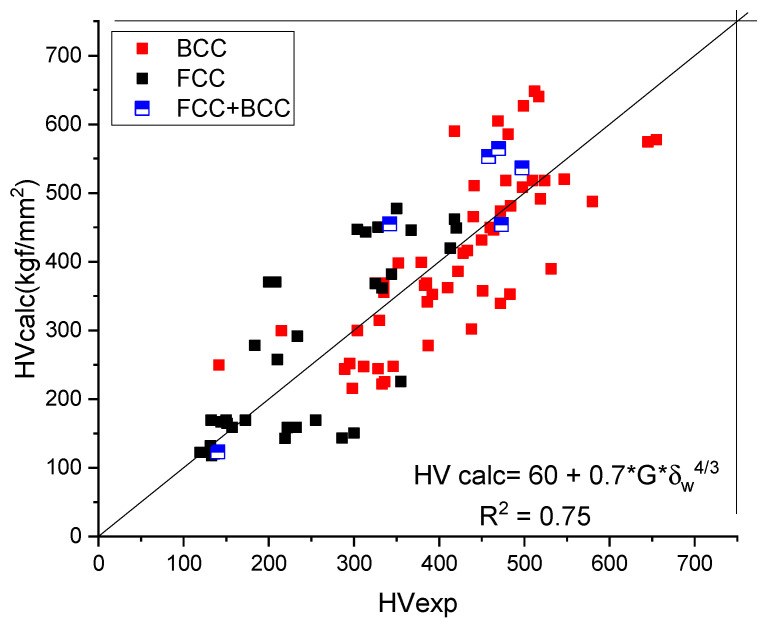
Comparison of the calculated and measured hardness values for BCC- and FCC-type HEAs.

In Table 4, a number of 106 HEAs were selected from the literature and the predicted hardness (Equation (24)) is compared to the measured one in Figure 6. We tried to find experimental hardness data that apply to single-phase alloys.

The goodness of fit, R^2^ = 0.74, is acceptable considering the scattering of the measured HV data and the number of approximations to express the formula for solid-solution hardening. Perusing Table 3, one can observe that no alloy sample with a shear modulus larger than 100 can be found. The atomic mismatch factor, *δw*, should be smaller than 6.5% for single-phase structures [25]. Applying Equation (24), we obtain HV = 900 kgf/mm^2^ as a theoretical upper limit for the hardness of HEA alloys.

Interestingly, serious deviation from the measured HV values, requiring addition of a much larger constant (150 ÷ 200 instead 60) signals the presence of precipitation hardening, and the alloy can no longer be considered as a single-phase material.

**Figure 7:**
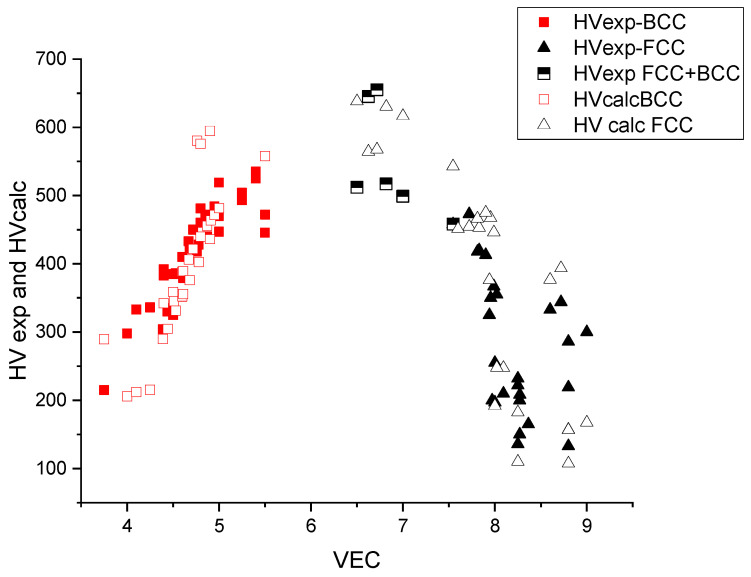
The hardness versus the average valence electronic concentration (*VEC*) for BCC- and FCC-type HEAs.

In Figure 7, the measured and calculated HV data are represented as a function of *VEC*. The maximum is approximately *VEC* = 6–7, which corresponds to the maximal, d = VEC − 2 = 5, uncompensated *d* electrons and hence to the maximal bonding strength (see Friedel model in [26,27]). The maximal hardness is approximately 600 kgf/mm^2^ and decreases both around *VEC* = 4 (refractory HEAs) and *VEC* = 8 (derivatives of FCC Cantor alloy).

From Figure 7, we can see that for small values of *VEC*, the structure is BCC and for large values, FCC. The structure is no longer single phase for intermediate VEC values. The extent of this double-phase (BCC + FCC) region is between 7.5 and 7.8, according to [28,29]. This double-phase region coincides with the maximum of the hardness as a function of VEC, which makes it difficult to interpret the hardness values in terms of solid-solution hardening only. The lack of BCC data around VEC = 6.5 is due to the fact that alloying the element of column 6 (Cr, Mo, W) with Fe and Co results in precipitation of intermetallic compounds like sigma phase [28] and it is difficult to find real single-phase HEAs with VEC = 6.5 in the literature.

**Table 4:** Comparison of calculated (Equation (24)) and experimental hardnesses of more than 100 single-phase high-entropy alloys.

Alloy		VEC	HVexp (kgf/mm^2^)	Gave (GPa)	δw (%)	HVcalc (kgf/mm^2^) BCC	HVcalc (kgf/mm^2^) FCC	HC calc (kgf/mm^2^) BCC + FCC	Ref.
Nb28.3Ti24.5V23Zr24.2	BCC	4.51	335	39.4	5.92	355.3	--	--	[30]
Nb22.6Ti19.4V37.2Zr20.8	BCC	4.60	352	40.4	6.43	398.1	--	--	[30]
Cr24.6Nb26.7Ti23.9Zr24.8	BCC	4.76	418	50.6	7.61	590.1	--	--	[30]
Cr20.2Nb20Ti19.9V19.6Zr20.3	BCC	4.80	481	50.35	7.59	585.7	--	--	[30]
Y25Ti25Zr25Hf25	BCC	3.75	215	22.99	7.58	299.6	--	--	pw
Ti33.33Zr33.33Hf33.34	BCC	4.00	298	35.01	4.00	215.6	--	--	pw
Ti30Zr30Hf30Nb10	BCC	4.10	333	35.27	4.10	222.0	--	--	pw
Ti25Zr25Hf25Nb25	BCC	4.25	336	35.67	4.13	225.4	--	--	pw
Ti20Zr20Hf20Nb20V20	BCC	4.40	392	37.32	6.12	352.4	--	--	pw
Ti25Zr25V25Nb25	BCC	4.50	385	39.57	6.10	368.7	--	--	pw
Ti20Zr20V20Nb20Ta20	BCC	4.60	410	45.01	5.45	362.2	--	--	pw
V25Nb25Mo25W25	BCC	5.50	472	89.88	3.06	339.5	--	--	pw
TiZrNbV	BCC	4.50	325	39.57	6.10	368.7	--	--	[31]
TiZrNbVMo0.3	BCC	4.61	379	44.14	6.03	399.1	--	--	[31]
TiZrNbVMo0.5	BCC	4.67	433	46.9	5.98	416.3	--	--	[31]
TiZrNbVMo0.7	BCC	4.72	450	49.46	5.93	431.6	--	--	[31]
TiZrNbVMo1.0	BCC	4.80	460	52.95	5.84	449.8	--	--	[31]
TiZrNbVMo1.3.	BCC	4.87	440	56.1	5.76	465.4	--	--	[31]
TiZrNbVMo1.5	BCC	4.91	472	58.02	5.70	473.5	--	--	[31]
TiZrNbVMo1.7	BCC	4.95	484	59.83	5.65	481.4	--	--	[31]
TiZrNbVMo2.0	BCC	5.00	519	62.28	5.58	491.5	--	--	[31]
TiZrNbV0.3	BCC	4.39	304	38.42	5.16	299.8	--	--	[31]
TiZrNbV0.3Mo0.1	BCC	4.44	330	40.28	5.21	314.7	--	--	[31]
TiZrNbV0.3Mo0.3	BCC	4.53	386	43.74	5.28	341.5	--	--	[31]
TiZrNbV0.3Mo0.5	BCC	4.61	383	46.89	5.33	365.6	--	--	[31]
TiZrNbV0.3Mo0.7	BCC	4.68	422	49.78	5.35	386.0	--	--	[31]
TiZrNbV0.Mo1.03	BCC	4.78	428	53.69	5.36	412.5	--	--	[31]
TiZrNbV0.3Mo1.5	BCC	4.90	464	59.27	5.33	446.3	--	--	[31]
NbCrMo0.5Ta0,5TiZr	BCC	4.90	469	58.34	6.98	604.7	--	--	[31]
NbTiVZr	BCC	4.50	335	39.57	6.10	368.7	--	--	[23]
AlCoCrFeNi	BCC	7.20	478.2	71.2	5.28	518.2	--	--	[32]
AlCoCrFeNi	BCC	7.20	509.6	71.2	5.28	518.2	--	--	[33]
AlCoCrFeNi	BCC	7.20	523.7	71.2	5.28	518.2	--	--	[34]
AlCoCrFeNiV0.2	BCC	7.03	546.8	70.1	5.36	520.2	--	--	[34]
AlCoCrFeNiV0.5	BCC	6.99	579.9	68.79	5.14	487.6	--	--	[34]
AlCoFeNi	BCC	7.50	441	60.69	5.88	510.8	--	--	[35]
Al18Co20Cr21Fe20Ni21	BCC	7.30	497.8	72.9	5.10	508.4	--	--	[36]
Al0.3HfNbTaTiZr	BCC	4.30	345.9	40.63	4.12	247.8	--	--	[37]
Al0.75HfNbTaTiZr	BCC	4.21	141.5	39.65	4.23	249.7	--	--	[37]
Al0.25MoNbTiV	BCC	4.97	450.9	57.03	4.51	357.5	--	--	[38]
HfMo0.25NbTaTiZr	BCC	4.47	387.1	44.96	4.27	278.0	--	--	[39]
HfMoTaTiZr	BCC	4.60	531.5	53.92	5.08	389.6	--	--	[40]
HfNbTaTiZr	BCC	4.40	328.3	41.36	4.01	244.4	--	--	[39]
HfNbTaTiZr	BCC	4.40	289.1	41.36	4.01	244.4	--	--	[41]
NbTaTiV	BCC	4.75	311.3	49.1	3.57	247.5	--	--	[42]
NbTaTiVW	BCC	5.00	438	70.4	3.30	302.1	--	--	[42]
NbTaVW	BCC	5.25	483	77.5	3.54	352.7	--	--	[42]
Hf0.5Nb0.5Ta0.5Ti1.5Zr	BCC	4.25	295	40.71	4.18	251.9	--	--	[43]
NbTiZr	BCC	4.33	289.1	37.82	4.27	243.4	--	--	[41]
FeCrNiCoAl2	BCC	6.50	512	66.66	6.69	648.2	--	--	[29]
FeCrNiCoAl1.5	BCC	6.82	517	70.36	6.36	640.3	--	--	[29]
FeCrNiCOAl1.25	BCC	7.00	499	72.47	6.11	626.6	--	--	[29]
fecrnicocual3	BCC	6.63	645	59.25	6.61	574.5	--	--	[29]
fecrnicocual2.8	BCC	6.72	655	60.1	6.57	577.6	--	--	[29]
FeCrNiCoAl2	BCC	6.50	512	66.66	6.69	648.2	--	--	[29]
FeCrNiCoAl1.5	BCC	6.82	517	70.36	6.36	640.3	--	--	[29]
FeCrNiCOAl1.25	BCC	7.00	499	72.47	6.11	626.6	--	--	[29]
--	--	--	--	--	--	--	--	--	
CoCrFeMnNi	FCC	8.00	132.3	84.72	1.58		169.1	--	[44]
CoCrFeNi	FCC	8.25	131.5	87.59	1.13		132.2	--	[45]
CoCrFeNiTi0.5	FCC	7.77	497.2	80.5	4.96		536.6	--	[46]
CoFeNi	FCC	8.99	119.5	77.7	1.11		122.5	--	[35]
CoFeNiV	FCC	8.00	233.5	68.7	3.25		291.5	--	[47]
Al5Co35Fe20Ni35Ti5	FCC	8.60	313.9	70.94	4.63		443.2	--	[48]
Co10Cr10Fe40Mn40	FCC	7.50	140.1	81.4	1.08		123.1	--	[49]
Co5(CrFeMnNi)95	FCC	7.81	150.9	86.43	1.51		164.8	--	[50]
Co10(CrFeMnNi)90	FCC	7.88	144.1	85.86	1.54		166.9	--	[50]
Co20(CrFeMnNi)80	FCC	8.00	149.9	84.72	1.58		169.1	--	[50]
CoCrFeMnNi	FCC	8.00	172.6	84.72	1.58		169.1	--	[51]
CoCrFeNi	FCC	8.25	156.9	87.59	1.43		158.8	--	[45]
Co0.5CrFeNi1.5Ti0.3	FCC	8.07	303.8	83.19	4.14		447.1	--	[52]
CoCu0.5FeNiTa0.1	FCC	9.16	183.6	73.05	2.97		278.3	--	[53]
CrFeNi2Ti0.3	FCC	8.18	328.3	83.31	4.16		450.2	--	[52]
--	--	--	--	--	--	--	--	--	
FeCrNiCoAlCu0,5	F + B	7.55	458	72.36	5.51			552.9	[54]
FeCrNiCoCu	FCC	8.80	133	79.2	1.03		117.7	--	[54]
FeCrNiCoTi0.3	FCC	7.95	350	84	4.35		477.5	--	[55]
FeCrNiCo	FCC	8.25	136	87	0.99		120.1	--	[55]
FeCrNiCoMo0.3	FCC	8.09	210	89.3	2.37		257.5	--	[54]
FeCrNiCoCu	FCC	8.80	133	79.2	1.03		117.7	--	[54]
--	--	--	--	--	--	--	--	--	
FeNiCrCuCo	FCC	8.80	286	79.47	1.35		143.3	--	[54]
AlCo0.5CrCuFeNi	F + B	7.72	473	66.9	4.94			454.0	[56]
CoCrFeNi-Pd1.5	FCC	8.72	344	73.4	3.96		381.9	--	[56]
CoCrFeNi-Pd	FCC	8.60	333	77	3.64		361.8	--	[56]
CoCrFeNiCu	FCC	8.80	219	79.4	1.35		142.9	--	[56]
Al0.5CoCrCuFeNi	FCC	8.27	208	72.74	3.88		370.4	--	[56]
CoCrCuFeNiAl0.5	FCC	8.27	200	72.74	3.88		370.4	--	[56]
CoCrFeNi	FCC	8.25	232	87.59	1.43		158.8	--	[55]
CoCrFeNi	FCC	8.25	222	87.5	1.43		158.7	--	[45]
CoCrNiCu	FCC	9.00	300	78.8	1.45		150.5	--	[55]
FeNiCrCuAl	F + B	7.60	342	66.2	4.99			455.1	[55]
AlCoCr0.5CuFeNi	FCC	7.99	367	63.3	5.07		445.9	--	[54]
AlCoCrCuFeNi	FCC	7.83	420	67.5	4.86		449.0	--	[56]
CoCrFeNi-Mn	FCC	8.00	255	84.7	1.58		169.1	--	[56]
CoCrFeNi-V0.3	FCC	8.02	355	84.2	2.17		225.6	--	[54]
CoCrFeNiCuAl0.5V0.6	FCC	7.94	325	69.85	3.98		368.4	--	[54]
AlCoCrCuFe0.5Ni	FCC	7.81	418	66.27	5.05		461.9	--	[54]
Co64Cr30W5C1	FCC	7.90	413	93.13	3.60		419.7	--	[57]
--	--	--	--	--	--	--	--	--	
Co10Cr10Fe40Mn40	F + B	7.50	140.1	81.4	1.08			123.1	[49]
Co5(CrFeMnNi)95	FCC	7.81	150.9	86.43	1.51		164.8	--	[50]
Co10(CrFeMnNi)90	FCC	7.88	144.1	85.86	1.54		166.9	--	[50]
Co20(CrFeMnNi)80	FCC	8.00	149.9	84.72	1.58		169.1	--	[50]
--	--	--	--	--	--	--	--	--	-
Al0.2CoCrFeNiTi0.5	FCC	7.57	469.8	77.45	5.33			564.7	[46]
CoCrFeMnNi	FCC	8.00	132.3	84.72	1.52		169.1	--	[58]
CoCrFeNi	FCC	8.25	131.5	87.59	1.43		132.2	--	[45]
CoCrFeNiTi0.5	FCC	7.77	497.2	80.58	4.96			536.6	[46]
CoFeNi	FCC	8.99	119.5	--	--		122.5	--	[35]
CoFeNiV	FCC	8.00	233.5	--	--		291.5	--	[47]

## 3. Conclusions

1. The metallic material was treated as a one-component plasma, where the positive ions with a charge equal to the metallic valence are embedded in the negative see of valence electrons. The results of coulomb crystal calculations concerning the shear modulus of neutron stars were adopted from the literature for metallic alloys.
2. The effective shear modulus can be calculated using only two parameters, valence, Z, and atomic radius, R_m_(Å), as:
  $$G_{eff}(GPa)=14\frac{Z^{2}}{R_{m}^{4}}$$
3. A formula for the cohesion energy was obtained using purely electrostatic considerations as a function of only two parameters, valence Z, and atomic radius R_m_(Å), as:
  $$E_{coh}(eV)=1.7\frac{Z}{R_{m}}$$

This formula permitted a new estimation of metallic valences based on the cohesion energy. Based on the strong correlation between the cohesion energy and the melting temperature, a simple formula can be derived for metallic valence:

Z = T_m_/500

4. We present formulas to estimate the hardness of pure elements from *E_c_* and *G*:
  $$HV_{calc}=0.37\cdot E_{c}\frac{Z}{V_{m}},\;HV_{calc}=1.67\cdot G,$$
  where [HV_calc_] = kgf/mm^2^, [G] = GPa, [E_c_]= kJ/mol, [R_m_] = Å, and [V_m_] = cm^3^.

A slight dependence on crystal structure was found in both cases.

5. Applying a number of simplifications and approximations to the solid-state strengthening formula of Maresca and Curtin, a simple formula was obtained to calculate the hardness of single-phase HEAs:
  $${\mathrm{HV}}_{\mathrm{calc}}=60+0.7\text{·}G\cdot \delta_{w}^{4/3}$$
  where [HV_calc_] = kgf/mm^2^ and [G] = GPa.

## Figures and Tables

**Table 2:** Comparison of the valences determined from Equations (5) and (8).

Metal	T_melt_	E_coh tabulated_	E_coh corrected_	Z-BM	Z-GK
	K	kJ/mol	kJ/mol		
Li	453.7	157.70	124.08	1.37	0.943
Na	371.0	107.50	102.98	--	0.949
K	336.4	89.889	93.860	--	1.07
Rb	312.6	82.208	87.650	--	--
Cs	301.6	78.088	84.640	--	--
Be	1560	319.73	432.76	2.60	2.49
Mg	923.0	145.64	259.86	2.60	2.10
Ca	1115	177.69	317.35	2.38	--
Sr	1050	164.24	330.80	--	--
Ba	1000	182.84	289.71	--	--
Al	933.5	327.28	263.75	3.07	1.90
Ga	302.9	270.61	85.930	--	--
In	429.3	243.08	119.89	--	--
Tl	577.0	181.58	161.90	--	--
Si	1685	445.78	502.00	3.26	--
Ge	1211	371.30	359.98	--	--
Sn	505.0	300.85	144.04	--	--
Pb	600.6	195.75	169.02	--	1.49
Sb	903.9	261.73	265.90	--	--
Bi	544.5	209.84	158.82	--	--
Te	722.7	209.35	215.95	--	--
Cu	1358	336.24	383.66	3.51	2.46
Ag	1234	283.48	348.75	3.65	2.54
Au	1338	368.03	379.05	4.68	2.75
Zn	692.7	129.47	194.97	2.83	--
Cd	594.0	111.00	167.07	2.86	--
Hg	234.3	62.418	65.700	2.60	--
Sc	1812	376.13	523.39	3.27	4.36
Y	1799	422.89	514.57	3.21	4.72
La	1193	430.82	335.56	2.81	3.17
Ti	1939	470.67	555.55	3.80	4.12
Zr	2125	608.70	606.87	3.97	4.98
Hf	2500	618.63	726.78	4.30	5.85
V	2190	510.87	626.85	4.08	4.36
Nb	2750	718.11	789.79	4.81	5.68
Ta	3258	781.33	934.05	5.16	6.74
Cr	2130	395.09	612.95	3.82	3.85
Mo	2896	656.66	838.90	5.56	5.76
W	3680	849.51	1068.5	6.11	7.36
Mn	1519	281.82	445.35	2.92	--
Re	3453	772.88	998.87	6.09	6.94
Fe	1809	412.93	528.81	3.85	3.31
Ru	2523	648.44	724.48	5.43	4.92
Os	3300	785.87	938.76	6.29	6.47
Co	1768	426.57	511.58	3.91	3.23
Rh	2233	550.80	643.48	5.37	4.35
Ir	2716	667.50	784.35	6.30	5.36
Ni	1728	427.83	497.76	3.93	3.12
Pd	1825	375.59	520.03	4.57	3.60
Pt	2045	563.43	584.70	5.62	3.29
Gd	1585	395.28	455.79	3.08	--
Dy	1682	290.01	485.93	--	--
Yb	1097	152.40	309.86	--	--
Lu	1936	427.17	559.49	--	--
U	1405	522.22	406.86	--	--

**Table 1:** The calculated shear modulus and cohesion energy using Equations (3) and (8) and comparison with the tabulated values.

Elements	*R_m_*	*Z_BM_*	*G*_accepted_	*G*_calc_	*E*_coh-accepted_	*E*_coh-calc_
	Å		GPa	GPa	eV	eV
		Equation (5)	Tabulated	Equation (3)	Tabulated	Equation (8)
FCC type		--	--	--	--	--
Al	1.43	3.02	26.1	30.56	3.39	3.59
Ca	1.97	2.45	7.4	5.617	1.84	2.12
Ni	1.25	3.85	78.6	86.34	4.44	5.25
Cu	1.28	3.45	47.7	62.51	3.49	4.59
Rh	1.34	5.41	147	125.8	5.75	6.84
Pd	1.37	4.58	52.1	82.46	3.89	5.66
Ag	1.45	3.57	29.8	41.00	2.95	4.20
Ir	1.36	6.43	221	172.0	6.94	8.06
Pt	1.39	5.62	63.7	120.0	5.84	6.89
Au	1.44	4.61	28	69.26	3.81	5.44
Pb	1.75	3.09	8.6	14.32	2.03	3.00
BCC type	--	--	--	--	--	--
Li	1.52	1.44	4.3	5.416	1.113	1.61
Na	1.86	1.48	3.5	2.588	1.113	1.35
K	2.30	1.45	0.9	1.050	0.934	1.07
V	1.31	4.03	48.1	77.34	5.31	5.23
Cr	1.25	3.82	90	83.65	4.1	5.19
Fe	1.24	3.81	81.9	85.94	4.28	5.22
Nb	1.43	4.79	37.6	77.38	7.57	5.70
Mo	1.36	5.53	118	125.6	6.82	6.91
Ta	1.43	5.10	70	87.40	8.1	6.06
W	1.37	6.07	151	147.1	8.9	7.54
HCP type	--	--	--	--	--	--
Mg	1.60	2.45	17.3	12.78	1.51	2.60
Sc	1.64	3.21	31.8	19.96	3.8	3.33
Ti	1.46	3.76	43.4	43.52	4.86	4.37
Zn	1.39	2.51	37.3	23.73	1.35	3.07
Y	1.80	3.20	25.4	13.66	4.37	3.02
Zr	1.60	4.09	36.8	35.51	6.25	4.33
Ru	1.34	5.56	163	136.0	6.74	7.07
La	1.88	2.74	14.1	8.442	4.47	2.48
Hf	1.58	4.26	55.8	41.02	6.44	4.59
Re	1.37	6.32	179	157.1	8.03	7.82
Os	1.35	6.64	214	185.4	8.17	8.35
Co	1.25	3.93	82.1	89.58	4.39	5.36

**Table 3:** Calculation of HV for pure elements from G and Ecoh.

Metal	HVmeas	Vm	ZBM	Ecoh-Corr	HVcalc	G	Hvcalc
FCC type	kgf/mm^2^	cm^3^		kJ/mol	kgf/mm^2^	GPa	kgf/mm^2^
			Tab		from Ecoh	Tab	from G
Al	16.7	9.99	3.022	263.8	30.68	26.10	43.59
Ca	17.5	29.19	2.450	317.4	10.25	7.400	12.36
Ni	63.8	6.59	3.847	497.8	111.8	78.60	131.3
Cu	36.9	7.09	3.447	383.7	71.75	47.70	79.66
Rh	124	8.3	5.414	643.5	161.4	147.0	245.5
Pd	46.1	8.85	4.581	520.0	103.5	52.10	87.01
Ag	25.1	10.27	3.570	348.8	46.63	29.80	49.77
Ir	206	8.52	6.427	784.4	227.6	221.0	369.1
Pt	54.9	9.09	5.622	584.7	139.1	63.70	106.4
Au	25	10.19	4.606	379.1	65.90	28.00	46.76
Pb	6	18.25	3.091	169.0	11.01	8.600	14.36
BCC type	--	--	--	--	--	--	--
Li	3	13	1.439	125.1	5.324	4.300	7.181
Na	2	23.76	1.478	103.0	2.464	3.500	5.845
K	1	45.94	1.453	93.86	1.141	0.9000	1.503
V	63	8.52	4.034	626.9	114.2	48.10	80.33
Cr	100.6	7.23	3.821	613.0	124.6	90.00	150.3
Fe	60.8	7.09	3.808	528.8	109.2	81.90	136.8
Nb	132	10.82	4.793	789.8	134.5	37.60	62.79
Mo	210	9.4	5.527	838.9	189.7	118.0	197.1
Ta	95	10.81	5.100	934.0	169.5	70.00	116.9
W	310	9.55	6.074	1068	261.4	151.0	252.2
HCP type	--	--	--	--	--	--	--
Mg	55	13.98	2.445	259.9	17.48	17.30	28.89
Sc	70	15.04	3.210	523.4	42.97	31.80	53.11
Ti	90.3	14.01	3.757	555.5	57.30	43.40	72.48
Zn	42	9.15	2.513	195.0	20.59	37.30	62.29
Y	20	19.88	3.199	514.6	31.85	25.40	42.42
Zr	90.3	14.01	4.086	606.9	68.08	36.80	61.46
Ru	230	8.2	5.555	724.5	188.8	163.0	272.2
La	49.1	22.58	2.740	335.6	15.66	14.10	23.55
Hf	176	13.41	4.263	726.8	88.87	55.80	93.19
Re	245	8.85	6.322	998.9	274.4	179.0	298.9
Os	309	8.45	6.645	938.8	283.9	214.0	357.4
Co	104.3	6.62	3.931	511.6	116.8	82.1	137.1

## Data Availability

The original contributions presented in the study are included in the article, further inquiries can be directed to the corresponding author.
